# An Enemy of the People—Tuberculosis and Natural Selection

**Published:** 1912-06

**Authors:** D. S. Davies

**Affiliations:** Medical Officer of Health, Bristol


### AN ENEMY OF THE PEOPLE—TUBERCULOSIS AND NATURAL SELECTION.

BY

D. S. DAVIES, M.D.,

*Medical Officer of Health, Bristol.*

---

A FULL belief in the desirability of a campaign to oppose the undue prevalence of tuberculosis is no bar to a dispassionate inquiry into the question whether we are to anticipate absolute extinction or only modified control.

On the one hand,[1] [2] with "infection" and "dosage" as the chief factors to be considered, we are promised that a comprehensive, co-ordinated scheme, comprising notification, the anti-tuberculosis dispensary, the sanatorium for early cases, the school for tuberculous children, the farm colony for working patients, and the hospital for advanced cases, will, if systematically taken in hand, completely eradicate the disease within a relatively short period.

On the other hand, we are reminded[6] that no two diseases agree exactly in the wideness of their distribution, or in the nature and extent of the "adaptation" by which, following the laws of parasitism, they maintain against heavy odds their existence as a species. We are reminded, further, that under the selective influence of disease persisting in the environment, certain races have already attained a condition of enhanced resistance over races not yet subjected to selection; and that attempts which only lessen risk of infection, without absolutely removing all risk, may in the event prove unwisdom for the race, however beneficial to the individual.

An early warning note to social reformers was sounded by Herbert Spencer,[10] who pointed out that a rational policy must recognise certain general truths of biology, and that it by no means follows, because a certain number of deaths by disease have been prevented, that so much pure benefit has been secured; for the net result of measures which tend to level down resistance, and to increase the numbers of the less resistant, may be merely the production of a somewhat larger number of a somewhat weaker race. The hope of the social reformer who trusts that the work of disease-control will be easier for future generations may be rudely shaken.

In favour of the anti-tuberculosis campaign, the notable and fairly steady decline since 1838, in the death-rate from this disease, which in 1895 showed a diminution of nearly two-thirds, is assumed to need only the stimulus of combined and continuous effort in order to blot out the disease.[2] This decline has been attributed by Ransome and others to "improved sanitary circumstances," and Newsholme has shown close correlation to exist between the fall in the phthisis death-rate, and such as conditions having influence on the well-being of a population the price of wheat, value of wages, institutional segregation, and so on. But Newsholme himself has pointed out that the decline from phthisis has shown no constant relationship to the decline of the general death-rate, and that whereas all countries have shown a decline in the general death-rate apart from phthisis, Ireland and Norway have varied from other countries in showing an increased phthisis rate.

Karl Pearson[3] also criticises the assumption that close correlation necessarily demonstrates causal influence, and proceeds to dissect the various factors involved in the declining rates. In a series of graphic charts he compares the decline in the general death-rate with the decline in the phthisis rate over three fairly equal periods between 1835 and 1910, and shows that while for males and females the general death-rate has continuously declined, with an accelerated rate for the middle period and a still greater acceleration for the later period, the rate for phthisis, though continuously declining, is doing so at an accelerated rate for the middle period, but at a considerably retarded rate for the third period, during which, indeed, it is tending to become stationary.

Further, on comparing the phthisis rate with the general death-rate as a percentage, it is found that while the second or middle period shows accelerated decline, the third period, that of greater knowledge and higher activity against tuberculosis, shows a relative increase, especially in the case of males, whereas if the tuberculosis campaign were exerting a causal influence on the phthisis rate, there should be increased relative improvement.

Pearson concludes that the curve of phthisis decline is not showing any tendency to finally cut the horizontal line, *i.e.* to achieve extinction, but rather to become parallel to it. Furthermore, we cannot suppose the steady increase as we go backwards to 1838 to have been prolonged indefinitely, otherwise total extinction of the race from phthisis would have happened. On the contrary, we may assume that there has assuredly been a time in this country when the phthisis rate was *rising*, just as it is now falling. We may, indeed, with the periodicity of other infective diseases in view, clearly realise the possibility of a typical "epidemic curve" in the frequency of phthisis, our years of registration experience coinciding with a down-grade.

Indeed, quite independently of any "sanitary" measures, it is fair to assume that, given a definite population and a definite number of infected persons to start with, under control of the ratio of non-immunes to the population at large (a ratio in its turn assumed to be dependent on the effect of natural selection through many years of ubiquitous tuberculosis), the percentage of infected persons tends either to rise or to fall, but in both cases to reach finally a certain more or less stable value, and to maintain itself for some time at this value. This appears to be a general truth in the case of widespread endemic disease, and in epidemic disease which has escaped control upon introduction.

*The ubiquity of tuberculosis.*—Immunity to disease can only be acquired as a racial character in the presence of selection; that is, the disease must persist in the environment. The degree of immunity attained by any given race will vary directly as the extent of the infection and the time during which selection has acted.

Tuberculosis in the Northern European races is so universal, that compulsory notification, while it reveals the clinical cases, gives no indication as to the full extent of its prevalence.

Careful *post-mortem* examination [6 7 8] has shown that unsuspected tubercle has existed in a large proportion of persons who ultimately die of quite other diseases. Bouchard gives a percentage of 75 from the Paris Morgue of persons, generally having died suddenly, in whom tubercular lesions, active or obsolete, were found. Ribbert, by systematic inspection, found that practically 100 per cent. of adults were affected. Nägeli laid down the principle that every adult is tuberculous, and Bertzke, from hospital experience, found in adults a percentage of 70 per cent. Brouardel further points out that in the majority of cases the lesions are not small disseminated foci, but large cicatrices, or wide, completely cicatrised cavities. It would appear that such ubiquity ensures the certainty of infection, in towns especially, once at least, perhaps many times, during life. Phthisis is, then, apparently self-curative in such races, even in advanced cases, and as phthisis accounts for only some 8 or 10 per cent. of the total deaths at the present time, a marked degree of resistance has somehow been attained.

This may be compared with the susceptibility in races where the disease has comparatively recently been introduced. Lawrason Brown [4] asserts that the morbidity and mortality among the American Indians from all forms of tuberculosis exceeds greatly those among the whites, and even the very high rates among the American negroes. The death-rate from pulmonary tuberculosis per 10,000 among the whites is approximately 17, among the negroes 40, while among the Indians it is 79.

In England and Wales in 1838 the rate was not quite 39, and this in 1895 had fallen to below 14.

*Immunity, Selection and Environment.* [6]—Racial immunity to disease must apparently result from the effect of natural selection acting upon the inborn variations occurring in individuals. It is improbable that the inhabitants of this country were more resistant to tuberculosis than the American

Indians when first the disease became prevalent. Resistance varied, and the most resistant survived to produce offspring whose inheritance of the characters of their parents, with variations towards more or less resistance, led to survival of the more resistant, and so on from generation to generation.

The process of selection is still going on, and immunity has not been attained by the race ; but though large numbers die who have varied in the wrong direction, the mean of the race has been raised.

Apparently what ought to happen is that complete immunity should be attained by the race if the selection is sufficiently stringent and lasts long enough ; but what does happen appears often to be that in place of attaining complete immunity, the race may develop an innate aptitude of acquiring immunity to the disease in so high a degree that the mortality is negligible in its effect upon the race. The example of measles may be quoted, and of malaria on the West Coast. [6]

Anything that diminishes the chance of infection generally in an immune race will tend to lower the racial standard ; hence, it is argued, [6] there is reason for doubt as to the wisdom of adopting general measures to lessen the risks of infection in the case of those diseases where it is impossible to hope that *all* risk of infection can be permanently removed. In the case of tuberculosis, it is doubtful whether anything more than a lessening of the risk of infection could be accomplished. We have already attained a high degree of immunity, and merely lessening the risks of infection without destroying them altogether would result in a lowering of the resisting power of the race, and might eventually end in disaster. In addition, by keeping alive for some time longer susceptible individuals at reproductive ages, one result is, as phthisis by no means lessens reproductive aptitude, to add to the population more individuals destined to succumb to infection.

Sandilands, [11] on the other hand, disputes the effective influence of natural selection in producing immunity, otherwise he claims that phthisis and plague over some thousands of years should not have failed to produce respectively immune human and rat populations. The failure, he argues, may be explained by the fact that the operation of natural selection against infectious disease invariably tends to defeat its own ends. The total extermination of susceptible individuals before they can reproduce themselves can only be attained by ensuring their exposure to infection *before the age when reproduction becomes possible.* If the first massacre falls short of total extermination, the course of subsequent epidemics is baulked by the presence of immune persons. Susceptible members of the community do not come into contact with infection, and their numbers consequently increase until epidemic prevalence again becomes possible. Under such conditions the action of natural selection becomes effectually hampered in producing a totally immune race.

The suggestion is that in the absence of discontinuous variations natural selection may produce periods of temporary immunity, to be followed by periods of enhanced susceptibility, but that it is powerless to produce an immunity which is absolute or permanent. In this view we arrive again at the concept of an epidemic wave.

The warning of the biologists is not intended, as Spencer[10] points out, to negative attempts at social amelioration, and Walker[6] specifically insists that consideration for the race is distinct from that for the individual. The effect of environment, of better conditions of life in general, of well-directed care and treatment, may be to preserve to maturity many whose existence is clear gain to the State. If the weakly never survived, Nelson had not won Trafalgar, and many whose resistance would normally suffice, deserve protection against adverse conditions which may turn the balance against them.

It is a clear gain to appreciate the weakness and strength of one's own side as well as the strength and weakness of the enemy, and more harm may result from despair at not realising the unattainable than from acceptance of inevitable limitations, while making a bold fight for the possible.

Already, to quote one instance, scarlet fever: in the eighties we misunderstood the message of causal organisms, and built our faith for its extinction on isolation and disinfection ; but these alone would not suffice, and the reaction caused many to speak disrespectfully of germs, as one might of the Equator. But though falling short of extinction, which may not be achieved, mitigation has resulted, and it is a clear gain to be able to record epidemics which, as in 1902–03, resulted in 115 deaths, in comparison with an epidemic in 1863–64 which resulted in 1,100 deaths in a city half the size.

Science, unlike politics, has no aim but ultimate truth. If we cannot do what we would, we must strive to do what we can; and in place of repining at our inability to stem an adverse tide, we may by joining boldness to discretion, steer many a frail craft athwart it into harbour.

REFERENCES.

1 *Report of the Chief Medical Officer of the Board of Education.* 1910.

2 Newsholme, *The Prevention of Tuberculosis.* 1908.

3 Karl Pearson, *The Fight against Tuberculosis, and the Death-rate from Phthisis.* 1911.

4 Lawrason Brown, *The Control and Eradication of Tuberculosis*, Ed. by Sutherland. 1911.

5 Osler, *The Principles and Practice of Medicine.* 1910.

6 C. E. Walker, *Hereditary Characters and their Mode of Transmission.* 1910.

7 Bertzke, *Über Haüfigkeit und Infectionswege der Tuberculöse. Tuberculosis*, Berlin, 1906, v., No. 4.

8 Brouardel, *Trans. Brit. Cong. Tuberculosis*, 1902, i.

9 Archdall Reid, *The Principles of Heredity.* 1905.

10 Herbert Spencer, *Study of Sociology.* 1881.

11 Sandilands, *Proc. Roy. Soc. Med.* (Epidem. Section), 1911–12, v., 49.

12 Cobbett, "Portals of Entry in Phthisis," *Ibid.* (Sect. Childr.), 1910–11, iv., 205.

